# The powerless bosses: the working day experience of bicycle messengers and its impact on their identities and wellbeing

**DOI:** 10.1186/s40359-024-02311-6

**Published:** 2024-12-31

**Authors:** Iben Østin Hjelle, Helle Nordrum, Julian-Werner Wentzeck, Ali Teymoori

**Affiliations:** 1 Department of Psychosocial Science, University of Bergen, Christies gate 12. 5015, P.O. Box 7807, Bergen, NO-5020 Norway; 2 Department of Philosophy, Berlin School of Mind and Brain, Humboldt-Universität zu Berlin, Berlin, Germany

**Keywords:** Bicycle messengers, Employment relations, Gig/platform economy, Group dynamics, Personal and social identity, Precarity, Working conditions, Wellbeing

## Abstract

**Background:**

Bicycle messengers in the online food delivery sector typically work on an on-demand basis, have digitally mediated relationships with their employer, and have very limited labor rights. In this study, we explore how bicycle messengers themselves experience their workday and how platform work influences their identity and wellbeing.

**Method:**

We conducted qualitative interviews with ten bicycle messengers working for Foodora and Wolt in Bergen and Oslo, Norway. We used reflexive thematic analysis to analyze the interviews.

**Results:**

We discerned five themes related to the dynamics of autonomy versus algorithmic control, the reduction of workers’ identity to the courier role, lack of collegial bonding among bicycle messengers, physical demands and risks of the job, and the transitory nature of courier work.

**Conclusion:**

The working experience of bicycle messengers is marked by a paradoxical sense of autonomy and powerlessness in the face of algorithmic control, a perceived sense of devaluation by others and its reproduction among their own colleagues, and competitive and at times hostile intra- and inter-group dynamics. We explore the implications of such working experiences for bicycle messengers’ wellbeing and identity within this newly evolving form of labor.

## Introduction

The entrepreneurial online food delivery business model, typical of the platform economy, promotes flexible labor and characterizes courier workers as being their own bosses. However, the types of employment relations and reimbursement that platform laborers including bicycle messengers experience have led some to believe that the platform labor is precarious work [1], with diverse ways of controlling, monitoring and surveilling the gig workers [2] and dramatic consequences for their wellbeing [1, 3]. Given the portrayal of couriers by the online platforms as independent yet facing precarious conditions, it is essential to consider couriers’ own perspectives on their working day experience. How do couriers perceive their job autonomy in the context of platform work and its algorithmic management? What resources do couriers have at their disposal to manage their work? How do they deal with the challenges and instabilities of gig work and their working conditions?

In addition, couriers typically engage in a freelance employment relationship on an on-demand basis, where their connection to their employer is mediated digitally through an online application. From a psychological perspective, we do not know much about how couriers experience their working day in a digitally mediated platform and how that influences their daily lives, as well as their personal, social and professional identities. Although sociological studies on the lived experience of gig work highlight the prevalence of constant surveillance in various forms in platform work [2, 4], and the entrepreneurial identity normalized through this digitally mediated work design [5], the psychological influence of such conditions remains relatively underexplored. What are the formative effects of couriers’ workday experiences on their personal, social and professional identities? How does the platform’s work design influence the way couriers relate to themselves, their colleagues, others and society at large?

Finally, app-based food delivery is becoming popular in Nordic countries [6], but the employment relationship it offers is unstable and insecure, hence differs significantly from the standard employment relationships common in the region [6, 7]. Such instability and insecurity in employment relationships, combined with algorithmic management of physically demanding and mentally taxing work, create conditions that put couriers’ safety and health at risk [3, 6, 8]. Thus, it is essential to understand how couriers perceive their working environment, navigate the challenges they face, and how they are influenced by the specific setup of work design in terms of its employment relationship and algorithmic management. We hope to provide evidence-based findings regarding the working experiences of couriers and their implications for workers’ wellbeing and identity in a relatively underexplored Norwegian context.

In the following sections, we first introduce the platform economy and platform labor, along with our psychological theoretical framework, covering two aspects of the working environment such as the job demand-resource model and various dimensions of personal, social, and professional identities, which will guide our empirical interview study. We then present our empirical case, focusing on Wolt and Foodora bicycle messengers in Bergen and Oslo, Norway. We argue that platform-based online food delivery companies in Norway, specifically Foodora and Wolt, have created a precarious working environment for their bicycle messengers, leading to significant consequences for their personal and social identities and wellbeing.

### Gig economy and gig work

The platform economy refers to the digital mediation of economic activity. In this new economic model, workers, often referred to as gig workers or platform laborers, engage in short-term, on-demand contractual employment typically in the form of freelance or part-time jobs [9]. This model reflects a significant shift in employment relations in comparison to traditional artisan and craft work or factory work [10, 11].

Several online food delivery platforms have emerged in recent years within the platform economy, including DoorDash, Postmates, Wolt, Grubhub, Foodora, and Deliveroo. The work design for online food delivery is that couriers are intermediaries between customers and restaurants through an online application. Customers place and pay for their orders, and the platform’s algorithm assigns the order to a nearby available delivery courier to pick it up and deliver it swiftly in return for a portion of the delivery fee [12]. Online food delivery platforms use diverse vehicles including bicycles, cars, and scooters. Although bicycles have been used for transport and delivery since the 19th century [13], their application for food delivery via online platforms is a relatively recent development.

Literature is somewhat divided on the implications of platform work for workers. On one hand, platforms are seen as offering advantages by flattening hierarchies, providing flexible work shifts, and offering autonomy and independence at work—often described as being one’s own boss. It is precisely the flexible working hours, relative autonomy to work at one’s own pace, the lack of directive control by supervisors, and very low entry requirements that are at times embraced by couriers [5], making platform work particularly appealing to students, migrants, those who want to have a second job, and those excluded from the more established labor market [14–16]. On the other hand, the employment arrangements and task-based structures are often criticized for exploiting cheap labor, offering minimal social protection, and creating precarious working conditions for platform workers [12, 15, 17, 18]. The employment relationship of freelancers as independent contractors is also contested for misclassifying workers to save costs, obfuscating the reality of gig workers’ working conditions, and perpetuating gendered and racialized low-income service workers [19]. It is perhaps no surprise that the demographics of platform-based delivery workers are predominantly young, foreign-origin males, students, or those who want to have a gateway into the labor market [14, 15, 19]. As a result, the work seems temporary for many of those who work as couriers [14, 16]. The lack of social protection, or the possibility of having sick leave and other benefits, makes workers’ conditions precarious since there is evidence of high exposure to risks such as road accidents, risky cycling to boost earnings for couriers, and cycling in areas that are not safe for bicycles [14, 15, 20]. Hence, there is a dual narrative surrounding platform workers’ experiences: the neoliberal optimism regarding platform flexibility versus the pessimism of critical literature about precarity of platform work [21].

From a social psychological perspective, a more effective approach to understanding the working experiences of platform workers is to analyze work design, assess how workers perceive their environment, and what psychological implications that might have on the workers’ identity and wellbeing. This account necessitates an emphasis on the dynamics between work design and various facets of workers’ agency and identity [22]. Key aspects of work design include the work management practices and the type of identity entrepreneurship that such work design constitutes.

Regarding the work design, the activities of freelancers or self-employed independent contractors are governed by algorithmic management [12, 18]. Deploying algorithmic management meant that the measurement, surveillance, and control of workers and all of their activities are under new automated operations. The automated operations include recording all work-related activities, directing work activities through automated and often opaque and non-transparent information processes, evaluating workers by recorded work activities and rating, disciplining workers through reward, punishment, and replacement [23]. Another aspect of the work design is the absence of a physical organizational space for workers, the technological infrastructure in the form of an application, a power imbalance in terms of information asymmetry, and obfuscated forms of performance management strategies by the platform providers [24]. That means workers’ connection to their employer is through a digital application which captures all their work activities and manages them by opaquely calculated performance ratings based on response rate to accepting or rejecting a task, their speed of accomplishing the assigned tasks, and customer ratings.

In addition to algorithmic management and steering workers’ performances through digital control, work design is structured to foster an entrepreneurial identity among gig workers [5]. That means that work design is such that gig workers are their own managers, disciplining their own labor power with algorithms that foster a rationality of hyper-meritocracy, efficiency, objectivity, and competitiveness among gig workers [5, 25]. The entrepreneurial identity also means gig workers must take charge of being organized, managing their commitment, investing in their own health, capabilities, skills, and training, and dealing constructively with their interactions, emotions, and risks [5, 22].

There seems to be a dual function of work design for individual identity. On the one hand, platform work design follows an entrepreneurial rationality that creates self-productive and autonomous workers with their own consent [5]. That means that the success of algorithmic management is partly due to recruiting the workers’ agency and securing their consent throughout the process. On the other hand, the work design might actually inhibit being autonomous due to algorithmic management of gig workers, digital surveillance, compensation based on task performance without benefits and paid leave, long working hours, low pay, fluctuation in demand, dependence on the rating system, competition between workers, pressure for timely delivery, and social isolation that prevents the formation of a sense of social and collective identity [14, 26]. These factors make the gig workers’ working conditions highly precarious [17, 20]. In fact, the algorithmic management can create conditions of fear, passivity, and frustration [23]. How does flexible yet precarious work influence and shape individuals’ identities? How do individuals experience digitally mediated platform work and algorithmic management of work tasks?

This paper extends the literature on lived experience of work by investigating the influence of such a working environment on individuals’ identity and wellbeing. It is likely that individuals might hold both beliefs of being autonomous yet powerless given the work design. In order to find out such implications of work design for individuals’ identities and wellbeing, we relied on two main psychological perspectives for our interview guide including the Job Demand-Resource model (JD-R) [27, 28] and Social Identity Theory (SIT) [29, 30].

### The conceptual framework: JD-R and SIT

Recently, Perrewé and colleagues [31] suggested that despite being a new form of labor process, gig workers can still be analyzed within the framework of the JD-R model [27, 32]. According to the JD-R model, workers’ health and wellbeing depend very much on the job demands and the resources they have at their disposal to satisfy those demands. In a quantitative study, Zhang et al. [28] investigated the JD-R model among delivery drivers in China and found that, consistent with the JD-R model, excessive job demands increase delivery drivers’ burnout, while job resources reduce burnout. However, the relationship between demands, resources, and psychological wellbeing can be influenced by various personal and situational factors, such as social and psychological capital, emotional labor, autonomy, and individual circumstances [31]. This is especially significant to explore since, in the context of platform work such as online food delivery, autonomy as a key resource in JD-R coexists with a sense of powerlessness in the face of algorithmic management [5].

In addition, JD-R postulates that there are consequences for individuals’ wellbeing in terms of resources and demands that individuals have at their disposal at work. For instance, lack of control over algorithmic management, constant surveillance, and career-path uncertainty often lead to burnout, depression, and negative emotions [4, 8]. Low pay also leads to high job dissatisfaction, frustration, and anxiety [15].

Moreover, the work design of online food delivery platforms has implications for individuals’ identities, a research area in platform work that is rather underdeveloped [22]. So far, previous studies on the lived experience of platform work show that individuals will develop an entrepreneurial identity [5, 22]. Entrepreneurial identity is just one aspect of the way individuals relate to themselves. The existing research already indicates that work design influences the intragroup dynamics such as the atomization of the workforce by design with strategies such as individualized performance assessment, induced competition, and receiving fewer offers when standing close to other couriers [5]. In addition, the workforce in platform sector, including the online food delivery sector, is far from homogenous. Platforms have a stratified labor market characterized by differential levels of inclusion and exclusion in the labor market, dependency on income from the platform, and the capacity to build individual and collective capital in terms of bargaining power, citizenship status, language proficiency, and work permit status of newly arrived migrant workers [16, 33], all of which influence the intra- and intergroup dynamics. There is also evidence that individuals might engage in resistance strategies by forming groups and creating new informal communication channels with their coworkers outside of routine work to connect, show solidarity, and build resilience [34].

Extending this line of personal, social, and professional identity as well as group dynamics from a psychological framework such as SIT [29, 30], we look into how platform workers form their identity in relation to themselves, their fellow gig workers, the platform, and society at large. This includes analyzing how individuals categorize themselves and other couriers into groups, to what extent they identify with those categories and groups, and how they envision the intergroup relations and group dynamics among couriers, toward the organization, and society [30].

### The current study

Despite extensive research on platform work in our neighboring disciplines, psychological studies examining perceived working environment, the formative effect of workday experiences on their personal, social, and professional identities, and the implications of platform work on workers’ wellbeing are still understudied. Employing a qualitative method is suitable to fill this research gap and is also crucial for gaining a deeper understanding of couriers’ working experiences from their own perspectives. In addition, there are few studies on couriers’ working experiences in the Norwegian context. In this section, we first clarify the focus of the current study, which is on bicycle messengers in Norway, and then explain our empirical design for the current study.

### Wolt and foodora in Norway

Norway, like other Nordic countries, has strong labor rights and a welfare system, but it is experiencing a neoliberal trend in its labor market, with the gig economy having a stronghold in the Norwegian economy as well. In terms of online food delivery, couriers from Wolt and Foodora dominate the urban landscape of Norway’s major cities. Foodora was introduced to Norway in 2015, followed by Wolt in 2018.

Wolt exclusively employs freelancers, meaning couriers are compensated solely based on the completion of on-demand tasks without additional benefits such as sick leave, paid holidays, or pensions. In contrast, Foodora offers two types of contracts for bicycle messengers: freelancers and permanent employees. Freelancers in Foodora have the same working conditions as their counterparts in Wolt, but Foodora’s permanent employees are required to work a minimum of 10 h a week. Despite offering only permanent contracts at the beginning, Foodora shifted its policy in Norway in 2019 to employ more freelancers, and as a result, the number of permanent workers has declined, with the company now employing mostly freelancers [6, 16]. The existence of two forms of contract in Foodora creates a more hierarchical setup where permanent employees often serve as rider captains, acting as immediate supervisors for Foodora’s freelance couriers. Unlike Foodora, which provides rider captains for freelancers to contact, Wolt has online customer service in the app as the sole point of contact.

Foodora and Wolt promote themselves by offering flexible work hours, advertising couriers as their own bosses. On their websites, they specify employment requirements, which include having a means of transport, a smartphone with data, and a valid ID or passport. While couriers may use various forms of transportation, this study specifically focuses on those using bicycles, as most couriers at the time predominantly used bicycles for deliveries. Given the diversity of our sample in other characteristics—such as employment type, demographic background, and the companies they work for—we chose to maintain homogeneity in the type of vehicle used, hence focusing mostly on bicycle messengers. Similar to other gig workers, bicycle messengers have contractual form of employment characterized by flexible working hours and less directive control, with workers contributing their own resources to complete the task at hand [9].

### Study design and questions

Using an exploratory interview method, this research investigates bicycle messengers’ own descriptions of their work experiences, the way they perceive the digitally mediated platform work, and the effects of these experiences on their identity and wellbeing. Extending the current literature with our psychologically informed conceptual framework, we examine how bicycle messengers’ working experiences influence their personal and professional identities, impact their relationships with colleagues, the organizations they work for, and society at large, and explore the group processes within the bicycle messengers’ workforce.

We designed our interview guide based on our theoretical framework and research questions. We first ask about the workers’ general perceptions of their working environment to understand how they view their working day and the digitally mediated nature of their work. Based on the JD-R model [27, 32] and the literature on the work design of platform work [5, 21, 24], we examine how bicycle messengers perceive the discrepancy between resources and demands and how they see their own labor within the particular design of online food delivery platforms.

Relying on the insights from SIT [30], we then ask questions regarding bicycle messengers’ social identity and intergroup relations. More precisely, we explore how bicycle messengers categorize their group membership, relate to their organizations, colleagues, and others at work, and how they compare themselves with others [29, 30, 35]. Finally, we evaluate how the work design influences workers wellbeing. Hence, based on the gaps in existing literature and our conceptual framework, we have the following three research questions:

- How do bicycle messengers perceive their working environment and experience their working day?
- How does the daily work experience of bicycle messengers affect their social identities in relation to their colleagues, profession, and society at large?
- What is the influence of work design and working day experiences on bicycle messengers’ wellbeing?

## Methodology

We use semi-structured interviews to collect data [36]. Based on our research questions, we developed an interview guide consisting of 16 questions and additional potential follow-up questions. We designed the interview guide in both Norwegian and English, since the bicycle messengers come from diverse backgrounds, and some might be new in Norway and have not yet learned the local language.

### Recruitment of bicycle messengers

We relied on the convenience and snowball sampling methods [37]. The snowball method of sampling is proven to be extremely useful for reaching vulnerable groups or hard-to-reach populations [38]. However, caution is warranted in using the snowball method since we also wanted heterogeneity in our sample [37]. We employed various recruitment strategies to reach bicycle messengers. Initially, we hung physical posters throughout the city, distributed flyers to bicycle messengers, and posted an ad on social media platforms. We also joined various Facebook groups on social media for bicycle messengers. Additionally, we actively approached bicycle messengers in the city center, easily identifiable by their uniforms, briefly informing interested individuals about the study and providing our contact details to reach out if they were interested.

We obtained three informants through the snowball method, and the rest were recruited through the convenience sampling method. Our sample included captain riders and freelancers, providing an opportunity to gain deeper insights into the group dynamics and various perspectives among bicycle messengers. Additionally, two of the informants had recently left the profession, but we considered them to be relevant informants as they had extensive experience as bicycle messengers. Each informant was compensated with a 300 NOK gift card for their participation.

### Participants

The sample consists of 10 participants (*n* = 10), all of whom were men, with majority being between 20 and 40 years old (Table 1). Nine out of ten informants were bicycle messengers in Bergen, and one was based in Oslo. Six of our informants went through online training that consisted of a few general videos, while four participants, all of whom were rider captains, went through physical training that involved follow-up by another rider captain. Half of our informants had university education; half were ethnically Norwegian, and the other half had a migration background.

**Table 1:** Description of the study sample

Informant	Migration Background	Age	Education	Type of employment relation	On-boarding training	Company
1	Migration Background	30–40	High	Freelance	Online	Both
2	Native	30–40	High	Employee – Rider captain	In-person	Foodora
3	Native	20–30	High	Employee – Rider captain	In-person	Foodora
4	Native	50–60	Intermediate	Employee – Rider captain	In-person	Foodora
5	Native	20–30	Intermediate	Employee – Rider captain	In-person	Foodora
6	Migration Background	20–30	Low	Freelance	Online	Wolt
7	Migration Background	20–30	High	Freelance	Online	Foodora
8	Native	20–30	Intermediate	Freelance	Online	Both
9	Migration Background	30–40	Low	Freelance	Online	Wolt
10	Migration Background	20–30	High	Employee	Online	Foodora

Notes: Both: Both Foodora and Wolt; Education: Low - primary school, medium - secondary, high - higher education. Employment: Employee has a permanent contract

### Data collection

The use of semi-structured interviews was crucial to exploring diverse experiences and perspectives. The interviews lasted between 40 and 60 min. Except for one digital interview, all interviews were conducted face-to-face by one of the researchers and were audio-recorded. The interviews were conducted over a period of six weeks. We manually transcribed the interviews using a slightly edited transcription method [36] that involved streamlining verbal expressions into written descriptions by removing some non-verbal cues, repetitions, and filler words. During the transcription process, we omitted all identifying information and replaced names with abbreviations or pseudonyms, ensuring that no participants could be identified in the transcripts.

### Reflexivity of the research group

Prior to the study, no members of the research team possessed specialized knowledge about the bicycle messenger profession, nor did they have any prior acquaintance with the informants. However, a brief conversation between one researcher and the first informant facilitated the latter’s participation in the study. The researcher conducting the interviews maintained a reflection diary, regularly updating other team members both verbally and by providing access to the diary. All team members met regularly during data collection and data analysis to reflect on the issues arising from the data collection and analysis process.

### Analysis

We employed reflexive thematic analysis for its flexibility and its capacity to deepen our understanding of participants’ experiences, thoughts, and feelings [39]. We began by familiarizing ourselves with our data during and after transcription. By reading the data several times, we gained a deeper understanding of the material (Phase 1). Furthermore, relying on semantic inductive coding [39], we developed a code list, focusing on meanings relevant to our research questions (Phase 2). Four interviews were coded collectively with most members of the team present to discuss the codes and reach consensus about the coding strategy and coding list. The remaining six interviews were coded individually; although the codes were regularly discussed in group meetings when needed. All team members met to identify recurring meanings and grouped the codes into potential themes, examining how they worked together (Phases 3 and 4). After several rounds of sorting and refining, we named and defined six robust main themes, each with two to four sub-themes (Phase 5). These themes were thoroughly refined to align with our research questions and were visualized through a thematic map.

## Results

**Fig. 1:**
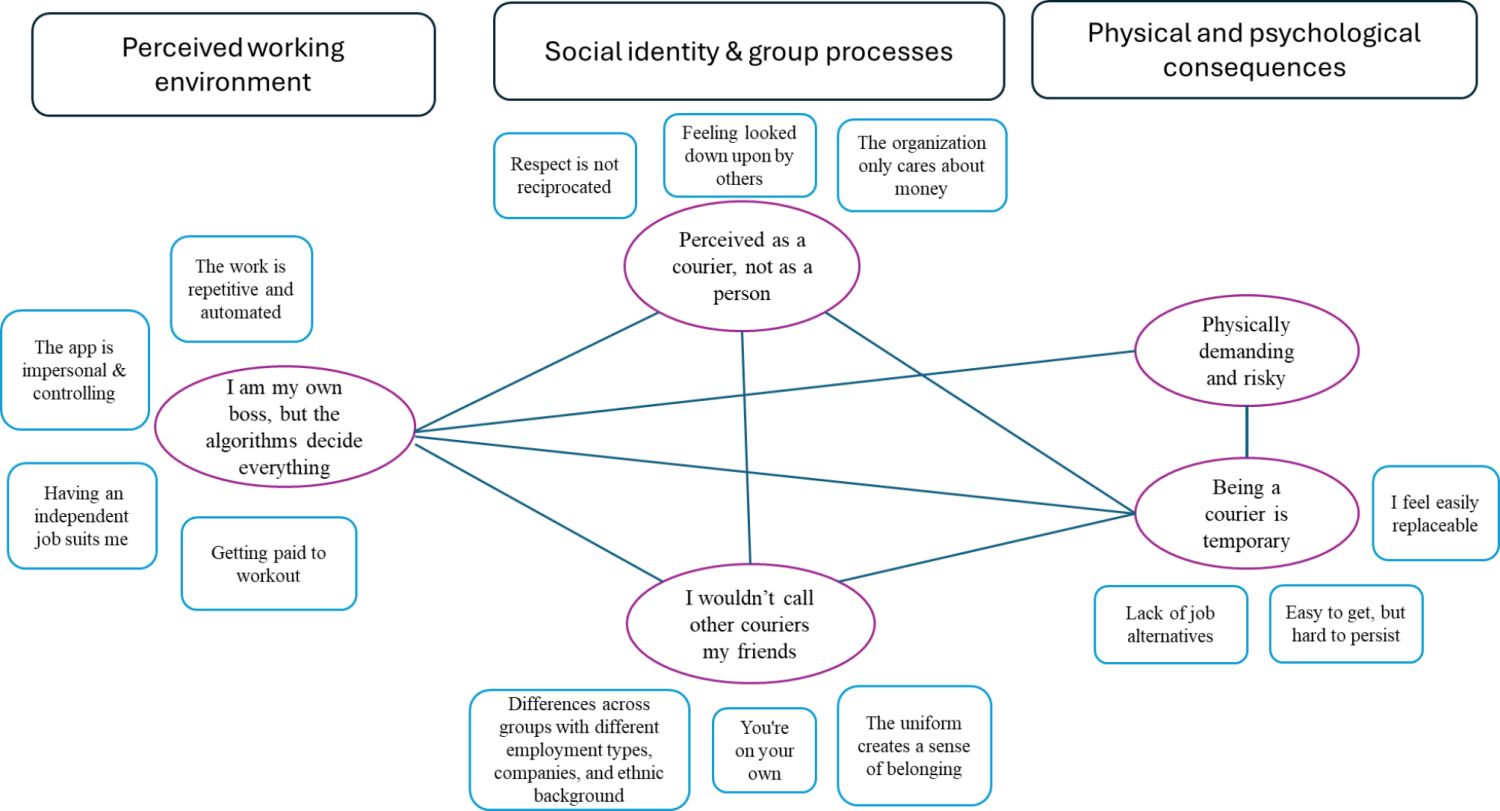
Thematic map of the study’s findings

We identified five main themes in our study in response to the three research questions we had. One theme provides insight into couriers’ working environment and the way they develop their professional identity: “*I am my own boss*,* but algorithms decide everything*”. In addition, two main themes highlight the group dynamics and social identity of the couriers: “*perceived as a courier*,* not as a person*” and “*I wouldn’t call other couriers my friends*”. Finally, two themes describe the consequences of courier work for individuals’ wellbeing: “*physically challenging and risky*” and “*being a bicycle messenger is temporary*”. Figure 1 depicts a thematic map of our findings.

### Bicycle messengers’ working day experience: I’m my own boss, but algorithms decide everything

While bicycle messengers may perceive themselves as their own bosses, they simultaneously experience limited control and autonomy in their daily work. Very frequently, our informants state that in the job they are their own bosses and have flexibility, autonomy, and the freedom to choose their own shifts: “*I am my own boss. I don’t feel stress of nobody. Whenever I feel stress I can just turn off the app and go home*” (I7, freelancer, Migration Background [MB]). However, at the same time, they did not feel that they have control over many things, such as not knowing the distance they have to go to arrive at the destination, the short amount of time they have to respond to a request, and the very limited opportunity to reject a request without consequences: *“.it’s the algorithms that decide*,* and as a courier I can’t do anything*” (I3, employee, native). The informants shared experiences related to orders they couldn’t reject, challenges with taking breaks during busy periods, and problems associated with the choice of the time of their work, showing a gap between the autonomy and flexibility they aspire to and actual working day experiences [40, 41].

As part of the aspiration for autonomy and flexibility in their work, several informants expressed how working independently is suitable for them. Bicycle messengers do not receive instructions from other people, and their working day is managed through an app, making it less likely for them to experience direct contact with a boss or a supervisor [9]. Being independent and the lack of interaction with others seem appealing to some couriers: “*It’s not uncommon for riders to experience anxiety*, e.g.,* social anxiety [.] It’s quite ideal to pick something up somewhere and deliver it to a friendly face at the other end and then run away*” (I4, employee, native). In addition, the promise of flexibility and freedom in the job generates expectations and aspirations among workers, as some informants find these qualities particularly well suited to their personalities: “*I understand myself better. in the future I am not going to have a team. This is not for my personality. I discovered these things. I prefer to work like this*” (I7, freelancer, MB).

The bicycle messenger work was also especially attractive to those who wanted to get some physical activity, expressing how it is like getting paid to work out: “*Yes*,* you really have such a handle on life. You get paid to work out and yes*,* to get out a bit*” (I2, employee, native). This was seen as a positive and motivating aspect of their workday. However, this was mostly the case for those who work part-time, the riders’ captains, and those who are not financially dependent on this job [42]:

“*I am someone who enjoys physical activity*,* being on the move*,* and seeing new things. I often feel a bit trapped if I’m in the same place all the time. That’s why it’s good for the soul to combine this job with something like office work*” (I1, freelancer, MB).

However, there were many occasions when bicycle messengers expressed how they do not have much autonomy and control over their work. For instance, in some cases, the option to take a break was deactivated during busy periods: “*That’s another thing*,* the algorithm […] will automatically put you into the next shift. If you planned your break*,* and it’s a big day*,* you’re not getting that break*” (I10, employee, MB). Moreover, many expressed frustrations with the way the app works, limiting their sense of control and autonomy and being punished through the rating system:

“*I was at 100% [in users’ rating] and I had five minutes left in my shift*,* and the app wanted to give me a double delivery. I wanted to go home*,* but that’s not an option*,* the app doesn’t take that into account […] I get home and I’m at 60% acceptance rate*” (I10, employee, MB).

Some bicycle messengers felt a very automated and mechanical working experience: “*So we end the conversation… and I often feel and think*,* ‘Is it a robot talking to me?’ The way they write*,* the way they respond*,* yet they have told me they are not robots.*” (I8, freelancer, native). Furthermore, the daily tasks of bicycle messengers are repetitive and offer limited learning opportunities: “*It’s quite repetitive*,* once you get the hang of it*,* you don’t learn anything new. You’re not challenged by anything new either*” (I3, employee, native).

Most of the bicycle messengers’ effort is directed toward achieving the best possible rating. Hence, the repetitive tasks and rating system have gamified the job for some of our informants, in which the app decides where you move, and you do your best to keep the rating high in that task: “*It’s just like a little mini game. Delivery one place*,* another place. Boom*,* boom*,* boom*,* boom*,* boom. Right. It’s not the worst*” (I10, employee, MB). Hence, the gamification seems to turn the monotonous nature of the work into a competitive challenge, increasing workers’ engagement with their tasks. For some others, repetitiveness meant that it requires minimal effort, allowing couriers to do something else on the side: “… *I ride every day. And at the same time*,* I can listen to podcasts*,* songs*,* and do whatever*,* calls and everything. I don’t feel like I’m doing a job*” (I7, freelancer, MB).

In general, working independently benefits bicycle messengers’ wellbeing by freeing them from directive control by someone else; however, their experiences reveal contradictions in that they are subjected to various means of control that challenge their independence. While they feel like their own bosses, they also experience a lack of control over the app and a sense of powerlessness in relation to the algorithms. The automated and repetitive pace of the work is perceived both negatively due to a lack of learning opportunities and positively due to its gamification, and it does not require much cognitive effort in performing the tasks.

### Social identity and intergroup dynamics among bicycle messengers

#### A) I wouldn’t call other couriers my friends

According to our informants, the bicycle messengers’ social relations on a daily basis are often limited to brief greetings when they meet. Such descriptions of the social dynamics among colleagues stem from many different factors, including the work design, heterogeneity of the workforce, group dynamics among bicycle messengers, the isolation of workers during work, and characteristics of their professional identity. It seems that couriers do not share a strong connection with their colleagues, as one states: “*I would say for myself*,* the standard for calling someone a friend is more than what I share with most of them [the couriers]*” (I8, freelance, native). This may indicate a lack of belonging within the organizational environment or collegial relationships, but certain factors inherent in the work design hinder meaningful interactions among workers.

The work design is such that bicycle messengers work primarily on their own: “*There is no boss*,* you work all alone*,* there are no colleagues*” (I6, freelancer, MB). This means that bicycle messengers do not have much social contact or any social arena to socialize: “*There is very little social contact*,* … if you don’t have other things going on in your everyday life like school or another job*,* then your social interactions at work are quite few*” (I3, employee, native).

When specifically asked about the chat group that some previous studies were pointing to [17, 34], our informants reported a change in dynamic of their social network, stating that it was the rider captains who had chat groups. The tendency to employ more freelancers meant not having those informal chat groups anymore:

“*I used WhatsApp*,* and initially*,* there were 100 members that were permanent employees. We shared tips and tricks*,* some shared jokes and daily stories*,* it was something that contributed to the sense of community. But as the number of [permanent] employees decreased*,* it died away a bit [.] You didn’t feel so lonely when you had the security and company in that group*” (I2, employee, native).

It seems that the identity of bicycle messengers initially take shape based observable factors, such as same uniform, which SIT would call it as situational factors to indicate common group membership [29, 30]. The uniform seems to increase the visibility of couriers in the city and acts as an identifier to other bicycle messengers, restaurants, and customers:

“*… we’re somewhat anonymous in the city*,* but we understand each other and can say hello and things like that. You recognize yourself in others because you do the same work*,* you look the same and you’re on the same team. It’s a bit like being at a football team with the same clothes*” (I1, freelancer, MB).

They could recognize and relate to other bicycle messengers by seeing them during their working day: “*At Wolt*,* everyone waves to each other*,* smiles at each other*,* everyone is usually very welcoming*” (I10, employee, MB). It is these informal meetings that create opportunities to share information and solidarity with their colleagues, especially concerning difficult situations related to incidents with customers and restaurants: “*These are things that bother me … I asked different people ‘did you have the same situation?’ and they said ‘yes*,* but let it go’*” (I7, freelance, MB). Thus, uniforms make the couriers recognizable to one another, which at times make them comfortable sharing information with each other, giving them a feeling that they are not completely alone in their working day.

However, while uniforms can foster a sense of belonging, they can also create divisions between groups and signal categorization in terms of ingroup and outgroup. Such categorization was more prevalent among the rider captains who held permanent contracts:

“*You’re a bit unsure when someone wears a Foodora helmet and a Wolt backpack*,* so who are you working for today? … Then it seems a bit sloppy that we don’t take things seriously or that it’s less professional now than when I started. Back then*,* those in uniforms were a team. … It’s cooler to be part of that community than in this somehow lax one*” (I2, employee, native).

This statement not only indicates the group division across the company that one works for (Wolf vs. Foodora) but also the division between the permanent employees vs. freelancers and the division between native couriers and couriers with migration backgrounds as the latter comprises most of the freelance workforce. In contrast to the nostalgia for a sense of community among the workforce expressed by permanent employees, freelancers referred to being a bicycle messenger as just a job and nothing more: “*We don’t really have a relationship. I deliver food*,* they send me the money. Finished. That’s it*” (I7, freelance, MB). For employees, this indicated a loss within the organization and a longing for the social community: “*They have a Foodora jacket and helmet*,* and pedaling on*,* cycling smoothly. That’s how we could have been. That’s how we were in the beginning. And that created something that still lives on in some of us*” (I4, employee, native). As another senior bicycle messenger with many years of experience indicated, the structural change to employ more freelancers changed the organizational environment for coworkers:

“*In the beginning*,* the environment was very nice. … At that time*,* everyone was a permanent employee*,* so we had a gathering point around The Blue Stone [in Bergen]*,* and got to know people well*,* always very nice people who greeted you when you cycled past them. So it was a nice environment to be part of. It changed a bit after they started freelancing*,* I think. … That was kind of the beginning of the end*” (I2, employee, native).

Thus, even with a digitally mediated employer, it would have been possible to create physical or virtual space to share and create a sense of community. Freelancing in a way takes away the social community aspect of the work and the work becomes truly a relationship between the worker and the app. It also introduces conflict among the workforce:

“*Because those who accept being a freelancer undermine everything we fight for. That’s because the company is then flooded with freelancers. So yes*,* freelancers are our enemy*,* and they destroy us all. Because they operate in open competition for tasks. We can be friends with them*,* there are a lot of good people there too*,* but they exploit the system and aim to make as much as possible in their own way when they can. So*,* it’s stupid*” (I4, employee, native).

The hostility between courier groups intersects various categories, including race (local vs. migrants), employment type (permanent vs. freelancers), and organizational affiliation (Wolt vs. Foodora), as permanent employees tend to be locals, while freelancers are predominantly students and migrants [42]. However, as reports of senior rider captains illustrate, the organization’s prioritization of the freelance scheme has had a major impact on the working lives of bicycle messengers, resulting in a weaker sense of community and cohesion.

#### Perceived as a courier, not as a person

It seemed to be a common experience for bicycle messengers when they meet customers and restaurants that they are reduced to their uniform and are not treated as a person. Some of our informants felt that they were not seen as a person by others:

“… *You become very impersonal*,* you just become Foodora. At the beginning*,* I was surprised by people at restaurants who would just say ‘hey you*,* Foodora’*,* and I was like ‘huh are you talking to me? My name is not Foodora’ […] you don’t really think much about the person behind it [uniform]*” (I1, freelance, MB).

In some cases, their reduction to uniform becomes internalized, as manifested in the way couriers refer to each other as Wolt or Foodora: “*You might say*,* for example*,* when you were cycling*,* you saw a ‘Wolt’*” (I6, freelance, MB).

The informants shared that they experienced a lack of respect in their encounters with restaurants. For instance, several informants reported that some restaurants do not treat them with respect: “*It’s a bit like that: ‘ahh you can’t even come in*,* just stand outside and wait in the rain’. Yes*,* there are some restaurants like that*,* and I don’t shop in them to this day…I think it’s so bad*” (I2, employee, native). Some informants also expressed being subjected to prejudice and discrimination, which was more common among migrant couriers:

“…*There was a girl who was the shift manager; she said ‘who do you think you are*,* knocking so loudly on the window’ … ‘Well*,* because you haven’t listened to me*,* so that’s why I’m knocking loudly’… They just complain and yell at me*,* ‘You don’t get your food’*,* she said*” (I6, freelance, MB).

The strain relationship of bicycle messengers with others was not limited to restaurant staff. They also felt that they were being judged and devalued by others. One source of devaluation is due to the profession being perceived as having low status: “*They judge you! You work as a Wolt*,* you’re not good. So*,* for example*,* if I’m an engineer*,* you have 100% respect for me*,* but if I’m a Wolt*,* no respect*,* so that’s how I feel*” (I6, freelance, MB). Being judged by others in this way may also impact how bicycle messengers relate to their own profession, which was a recurring theme in several of the interviews: “…*There’s nothing I can be proud of*,* ‘I work with Foodora’*,* you know. My dad’s just like ‘oh*,* you do…*’“(I1, freelance, MB).

The feeling of being devalued is due to the organizational environment and working conditions. Several of our informants feel that the organization cares only about money and bicycle messengers have a sense of being devalued: “*They’re managed by Delivery Hero [the company that owns Foodora]*,* which is a very shitty company when it comes to treating people with respect. They do whatever it takes to make as much money as possible at any given time*” (I4, employee, native). Hence, they regard the organization as business-oriented: “I *think they care more about the business than anything else*” (I7, freelance, MB).

### Work design influence on couriers’ wellbeing

#### A) being a bicycle messenger is temporary

Nearly all our informants did not envision a future as bicycle messengers for various reasons, as one of our informants stated: “*That’s the nature of the job. It’s not a job you hold permanently*,* very few people stay in it for a long time*” (I5, employee, native). One significant factor is the unpredictability of income, creating a high level of uncertainty and insecurity in the daily lives of bicycle messengers: “*I don’t know what’s going to be like next year*,* it could be bad*,* for example*,* earning 500NOK per day. That’s not enough money. It’s not a kind of viable future job*” (I6, freelance, MB). As a result, the inherent instability and financial uncertainty associated with it lead most couriers to see the job as temporary.

The job was considered temporary because the bicycle messengers often chose this work due to the lack of other opportunities: “*the reason I chose to work as a food delivery worker is because I have no choice*” (I7, freelance, MB). This was especially the case for migrants and students. Despite having a high level of education, some experienced difficulties entering the Norwegian labor market. The job was an entry point for several of the informants and a source of income on the way to another job: “*I applied for other jobs before that*,* but they were retail jobs. I was 18 at the time*,* they prefer students or slightly older people*,* with experience. Whereas with Foodora*,* everyone really gets a chance there*” (I3, employee, native). The entry requirement is very easy, making it a good temporary solution for those who are on the lookout for a job [14].

Despite being easy to obtain, people do not remain in the job for various reasons. In addition to the uncertainty and insecurity of income, we found that a lack of labor rights, such as sick leave and paid holiday, can make the job challenging in the long run: “*One month ago*,* I was sick for almost two weeks. I received nothing*,* so it was hard for me. But this is temporary. I am thinking about the same job*,* the same things*,* but with more rights*” (I7, freelance, MB). In addition, several informants felt that being a bicycle messenger was not suitable for everyone due to the weather conditions in Norway and the physical strain: “*You must have a certain stamina to continue even in bad weather and under poor conditions. You have to do what you have committed to*” (I5, employee, native). It’s also a physically demanding job and thus difficult to keep it for a long time: “*It’s physically demanding. The first month is tough. You think you’ve cycled up Bergen’s hills before (laughing)*,* but then you taste blood. That’s when some people give up*” (I2, employee, native). Consequently, few people endure these conditions for long.

Finally, the perceived temporary nature of the work seems to be part of the work design, as several informants felt that they were easily disposable: “*I don’t have any faith in the company when they say ‘don’t worry we like you here*,* we’ll change things. I think it’s instantly gonna be like*,* ‘you don’t want to go here*,* bye-bye’*” (I10, employee, MB). Such a feeling conveys a message that the company does not care about creating long-term relationships with its employees.

#### B) physically challenging and risky

Physical activity constitutes a significant part of a bicycle messenger’s working day, rendering the job both physically demanding and hazardous. Inadequate or nonexistent bicycle lanes and bad weather increase the risk of accidents and injuries for cyclists:

“*Being able to cycle on the kind of roads and conditions we have at times without getting hurt is something I’ve learned over time after injuring myself a few times. I’m probably the Foodora cyclist or courier in Norway who has delivered the most orders with a broken collarbone*” (I4, employee, native).

In addition, the job’s challenges extend beyond accidents; cycling in bad weather, up hills, and over long distances is very strenuous, with some informants reporting physical pain in their backs and knees. Bicycle messengers can become agents of their own exploitation by choosing to work very long shifts—some informants work as much as 12 h a day—primarily to earn enough money to make ends meet [43]. This is especially true for freelancers who are paid per delivery, with several reporting physical exhaustion after a day of work: “*Well*,* it’s a physical job*,* so if you work a lot*,* you’re tired in your free time . you earn enough*,* but you are very tired the next day*” (I5, employee, native). The autonomy that was consistently reported as a motivation in their work meant that they chose to work so much that they sometimes wore themselves out and injured themselves.

The rating system and the competition could also lead to dangerous situations. Bicycle messengers often prioritize their rating over their own safety, as a slight delay might lead to a bad rating. One very conscientious courier stated that, unlike others, he waits at red lights to obey the rules; however, then he rides his bicycle as fast as he can compete with other couriers: “*So we have to compete against each other and cycle fast. I made up for it by riding fast; I had like proper training and can ride really fast to make up for the time lost waiting at red lights*” (I1, freelance, MB).

These findings show that flexibility does not necessarily mean autonomy for workers but might turn them into agents of their own exploitation to work long hours and expose themselves to risk to earn enough and achieve a good enough rating.

## Discussion and conclusion

In our study, we explored how bicycle messengers perceive their working environment and the way in which such working environments have an impact on their identity, group dynamics, and wellbeing. Based on qualitative interviews with bicycle messengers, we have generated five themes pointing to a paradoxical sense of autonomy and powerlessness in the face of algorithmic control, a perceived sense of devaluation by others and its reproduction among their own colleagues, competitive and at times hostile intra- and inter-group dynamics, and health and wellbeing consequences of work for couriers.

Bicycle messengers seem to have a contradictory sense of their identity, where they experience both autonomy and powerlessness. The autonomy stems from the flexibility of the work and the absence of direct supervisory control [5, 9], a condition characterized as being one’s own boss, which promotes an entrepreneurial identity [22], whereby workers become the agents managing their own labor through a rationality of hyper-meritocracy, efficiency, and competitiveness [5, 25]. However, in practice, bicycle messengers have limited control over many aspects of their work due to various control mechanisms in place, including a rating system, asymmetrical access to information related to work tasks, and performance management strategies deployed by the platform [24].

Autonomy that is a resource in the JD-R model [27, 32] remains superficial, which should lead to lower engagement. However, as our informants noted, the platform employs strategies to increase worker engagement, such as gamifying work tasks [22, 24]. This finding aligns with existing literature that the online food delivery platform’s narrative of job flexibility and the idea of being one’s own boss coincide with lower income, longer working hours, and increased job insecurity for workers, issues that diminish a sense of autonomy for workers [8, 40, 41] and create a precarious working condition [15, 17, 40]. Many informants perceived having autonomy and flexibility, only to find themselves self-exploiting and working long hours [43].

Group dynamics among bicycle messengers led to two key findings. First, couriers reported experiencing a sense of disrespect from society and family due to the perceived low status of their job, as well as feelings of being disposable labor to the organization, and instances of discrimination and prejudice at work. Hence, according to SIT [29], couriers might develop a sense of being marginalized and stigmatized due to such experiences of disrespect. Second, there seems to be hostility between bicycle messengers that intersects various categories such as race (native vs. migration background), employment type (permanent vs. freelancer), and organizational affiliation (Wolt vs. Foodora). The heterogenous nature of the workforce and the hierarchy among Foodora’s workforce seem to create a condition where courier workers easily categorize themselves into different groups [30], feeding into this hostility between them and preventing a sense of solidarity among couriers. But as reports of senior rider captains illustrate, the organization’s prioritization of the freelance scheme has had a major impact on the working lives of bicycle messengers, resulting in a weaker sense of community and cohesion. Hence, the work design also prevents a sense of solidarity among courier workers [5].

This contradictory sense of being autonomous yet powerless, which we refer to as powerless bosses, combined with an atomized workforce and rather tense intergroup dynamics can lead to a condition that one can describe as anomie—i.e., a perceived sense of deregulated and disintegrated environment [44–48]. When the organizational environment is dominated by very high economic rationality and deteriorating working conditions, it can result in organizational anomie [49]. The anomic condition can have severe consequences for individuals’ personal and social identities [44, 45]. The damaged identity, erosion of a sense of community and solidarity, and the diminished chance for learning new skills can further complicate individuals’ ability to navigate their roles within both the labor market and society as a whole.

Finally, consistent with previous studies, working as bicycle messengers in the platform economy has significant implications for couriers’ physical and mental health [50]. Many bicycle messengers do not have a stable income or access to the established labor market, rendering their jobs temporary and contributing to insecurity and uncertainty regarding their career paths [16]. On the other hand, despite being easy to get, many bicycle messengers struggle to sustain their livelihood due to the physical demands of the job and the frequent accidents and risks [8, 50].

In general, the working experience of bicycle messengers seems to constitute several contradictions. Couriers seem to experience being their own boss but feel powerless in terms of the lack of control over their work. They also expressed frustration over being perceived merely as bicycle messengers rather than as individuals, a view they paradoxically apply to their coworkers as well. Moreover, workers often prioritize ratings and competition over their own safety, even while acknowledging the folly of such actions. Interestingly, an informant reported cycling very fast to circumvent risky behaviors like crossing red lights. Lastly, although many bicycle messengers recognize that the organizational business model exacerbates their working conditions, they frequently blame their colleagues, leading to complex dynamics of in-group and out-group formations among bicycle messengers. Many of the contradictions reported by our informants stem from the work design of online food delivery platforms [40]. For instance, these platforms offer job flexibility for choosing one’s working time but have a comprehensive digital infrastructure, such as a rating system and deactivation of break option at times, to monitor, control, and discipline workers. Such a work design, which includes several contradictions, may have implications for employees, including the risk of high organizational anomie and its severe outcomes for individuals’ identity and social and political integration—an issue that can be explored in future studies.

It is important to acknowledge the limitations of our study. We focused exclusively on bicycle messengers, as the majority of the courier workforce used bicycles; however, many also utilize other modes of transport, such as e-bikes and e-scooters, which may not find the job as physically demanding as bicycles messengers do. Additionally, our sample consisted solely of male couriers. While the courier workforce is predominantly male, there are women couriers, and it would be valuable to understand their perspectives on the working environment and whether gender serves as another axis of intergroup differentiation that we may have overlooked in our study.

Finally, it is important to note that the work design did not go unchallenged by the workers. During coding, it was observed that informants described various personal strategies to cope with job demands. These strategies were not systematically analyzed, as they were beyond the scope of the current research but they warrant further investigation in future studies. For instance, some informants were contemplating the ideal and proper job based on their experiences [17], and at times, expressed solidarity with their coworkers [34], all of which are signs of everyday resistance that can be studied further.

## Data Availability

An anonymized version of the interview transcript can be made available from the corresponding author for research purposes.
